# Left ventricular extracellular volume measurements in repaired tetralogy of Fallot patients

**DOI:** 10.1186/1532-429X-18-S1-P164

**Published:** 2016-01-27

**Authors:** Sara K Swanson, Jimmy C Lu, Sunkyung Yu, Maryam Ghadimi Mahani, Prachi P Agarwal, Adam L Dorfman

**Affiliations:** 1 Pediatric Cardiology, University of Michigan, Ann Arbor, MI USA; 2 Radiology, University of Michigan, Ann Arbor, MI USA

## Background

Although cardiac magnetic resonance (CMR) imaging is routinely used for evaluation of patients with repaired tetralogy of Fallot (TOF), the role of extracellular volume (ECV) measurement, a marker of diffuse myocardial fibrosis, has not been studied extensively in this population. This study aimed to describe left ventricular (LV) ECV measurements in subjects with repaired TOF, and to evaluate for associations with surgical factors, ventricular size and function, and aortic distensibility.

## Methods

All patients ≥ 14 years old referred for clinical CMR with contrast from November 2014 to August 2015 were eligible for inclusion. Patient and surgical characteristics were reviewed. In addition to the standard clinical CMR protocol on a 1.5 Tesla scanner (Philips Ingenia), a modified look-locker inversion recovery sequence was acquired in end-systole in a midventricular short axis plane before and 15-20 minutes after 0.2 mmol/kg gadolinium injection. LV ECV was then calculated from pre and post-contrast T1 times of the myocardium and blood volume, and hematocrit. Ascending aortic distensibility was calculated from phase contrast images. To determine correlations between ECV and patient/surgical factors and CMR-based measures of cardiovascular and aortic function, Pearson or Spearman correlation coefficients or t-tests were used as appropriate.

## Results

Twenty (median age at CMR: 24 years old, 60% male) out of 23 eligible subjects were recruited (2 declined, 1 excluded due to incomplete MRI). All patients had undergone complete TOF repair (median age: 1.2 years), with 4 patients having a history of prior palliative shunt (20%), and 8 (40%) additionally having undergone surgical or transcutaneous pulmonary valve replacement (PVR). The mean LV ECV was 0.27 ± 0.03. Age (p= 0.90) and surgical factors such as history of palliative shunt (p= 0.10) or PVR (p= 0.78) were not associated with ECV. The mean indexed LV end-diastolic volume was 90 ± 15 mL/m^2^ (z-score: -1.3 to +2.7), the mean LV ejection fraction was 53 ± 4% (z-score: -4.8 to -1.0) and the mean indexed LV mass was 57 ± 10 g/m^2^ (z-score: -2.4 to -1.1). None of these were associated with ECV. The mean aortic distensibility was 3.5 ± 1.6 x 10^-3^ mm Hg and was not correlated with ECV (p=0.86). Lower aortic distensibility indicative of greater aortic stiffness was associated with older age at CMR (r=-0.75, p = 0.0002), older age at TOF repair (r=-0.71, p = 0.0005), increased aortic regurgitant fraction (r=-0.49, p = 0.03), larger ascending aorta diameter (r=-0.71, p = 0.001), and increased indexed LV mass (r=-0.62, p = 0.004).

## Conclusions

In this small cohort of repaired TOF patients, LV ECV measurements were slightly higher than published reference values, suggestive of increased diffuse fibrosis, but did not correlate with surgical factors, ventricular size and function, or aortic distensibility.

